# The Research Progress of Exosomes in Osteoarthritis, With Particular Emphasis on the Therapeutic Effect

**DOI:** 10.3389/fphar.2022.731756

**Published:** 2022-03-02

**Authors:** Shang Xian Bo, Wang Chen, Liu Chang, Yu Hao Ran, Guo Hui Hui, Zhu Ya Kun, Xie Wu Kun, Fan Hai Tao, Cheng Wen Dan

**Affiliations:** ^1^ Anhui Medical University, Hefei, China; ^2^ Second Hospital of Anhui Medical University, Hefei, China; ^3^ Armed Police Corps Hospital of Anhui Province, Hefei, China; ^4^ Fuyang Hospital of Anhui Medical University, Anhui, China

**Keywords:** exosomes, extracellular vesicles, osteoarthritis, chondrocyte, extracellular matrix, treatment

## Abstract

Exosomes participate in many physiological and pathological processes by regulating cell-to-cell communication. This affects the etiology and development of diseases, such as osteoarthritis (OA). Although exosomes in the OA tissue microenvironment are involved in the progression of OA, exosomes derived from therapeutic cells represent a new therapeutic strategy for OA treatment. Recent studies have shown that exosomes participate in OA treatment by regulating the proliferation, apoptosis, inflammation, and extracellular matrix synthesis of chondrocytes. However, studies in this field are scant. This review summarizes the therapeutic properties of exosomes on chondrocytes in OA and their underlying molecular mechanisms. We also discuss the challenges and prospects of exosome-based OA treatment.

## 1 Introduction

Osteoarthritis (OA) is a prevalent degenerative joint disease that affects more than 500 million people worldwide (GBD 2019 Diseases and Injuries Collaborators, 2020) and is a major cause of chronic disability in the elderly (Tang et al., 2016). OA is caused by chondrocyte inflammation and apoptosis, which is the result of many factors. This leads to articular cartilage destruction, subchondral bone remodeling, and osteophyte formation (Ma et al., 2019). Chronic pain, motor dysfunction, and disability caused by OA not only significantly reduce the quality of life of patients (Hunter and Bierma-Zeinstra, 2019) but also bring a huge economic burden to individuals and society. The current treatment for early OA includes non-drug therapies such as exercise, weight loss, and physical therapy (Bannuru et al., 2019) and anti-inflammatory and analgesic drug therapy, such as nonsteroidal anti-inflammatory drugs and tramadol (Peat and Thomas, 2021). Advanced stage patients with severe dysfunction require joint replacement surgery, which often results in poor function, infection, prosthesis loosening, and other complications (Cottino et al., 2017; Kearns et al., 2018; Caron et al., 2021) that ultimately lead to pain and disability. Because of the complex pathogenesis of OA, there are no satisfactory treatments to cure or delay the pathological progression of OA (Butterfield et al., 2021).

In recent years, researchers have begun to explore the potential benefit of exosomes for OA treatment. Exosomes are a subtype of extracellular vesicles (EVs) with a diameter of approximately 40–160 nm (Kalluri and Lebleu, 2020). They may originate from body fluids, such as blood, saliva, plasma, urine, and amniotic fluid, and from various cell types, such as fibroblasts, epithelial cells, hemocytes, adipocytes, neurons, stromal cells, tumor cells, chondrocytes, and mesenchymal stem cells (MSCs) (Kim G. B. et al., 2021). The function and biological properties of exosomes depend on their cargo (proteins, lipids, and nucleic acids). In a pathological microenvironment, exosomes released by cells may promote disease progression, whereas those released by therapeutic cells may contribute to the treatment of disease. Exosomes derived from therapeutic cells can regulate the proliferation, apoptosis, and inflammation of chondrocytes and promote extracellular matrix (ECM) synthesis (Tofino-Vian et al., 2018; Qi et al., 2019; Sun et al., 2019; Li et al., 2020).

In this review, we summarize the various roles of exosomes on chondrocytes in OA, including the regulation of chondrocyte proliferation, apoptosis, inflammation, and ECM synthesis, as well as the underlying molecular mechanisms.

## 2 Biological Properties of Exosomes

Exosomes are a subtype of EVs. EVs are small membranous vesicles released by cells into the ECM. EVs participate in various biological processes, including cell communication, migration, angiogenesis, and tumor cell growth. EVs exist in various body fluids and cell supernatants and stably transport important signaling molecules. EVs may be categorized as exosomes, microvesicles, and apoptotic bodies based on their size, expression of membrane markers, and biogenesis (Kim G. B. et al., 2021). Of these, exosomes exhibit higher stability, inclusion, and better performance, which are of particular interest to researchers (Samanta et al., 2018; Gurunathan et al., 2019).

**FIGURE 1:**
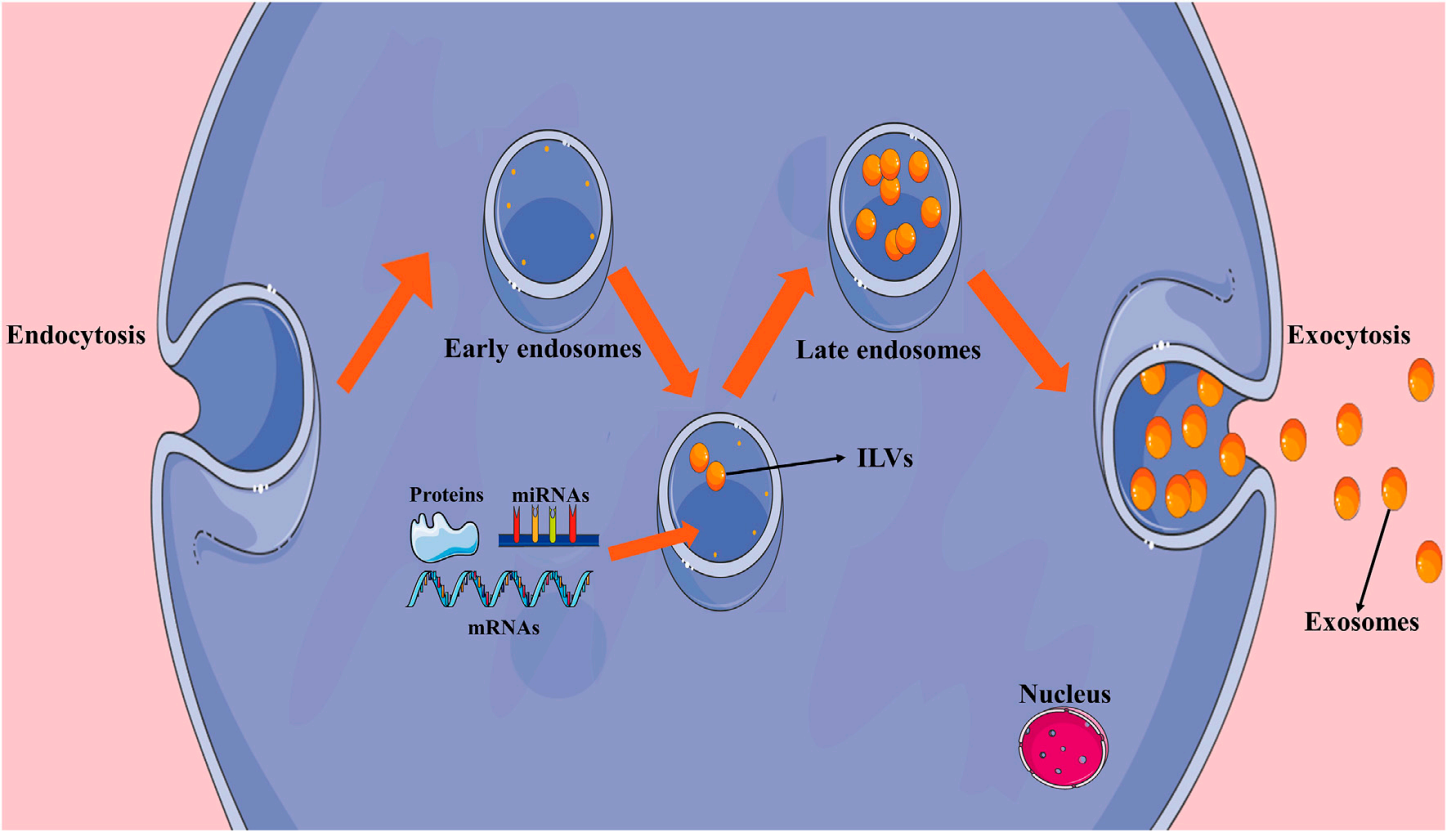
Formation of exosomes. Early endosomes are formed by endocytosis and intralumenal vesicles (ILVs) are formed through the internal budding of the endosomal membrane. After early endosomes transition to late endosomes, ILVs are released extracellularly as exosomes.

Exosomes are generally formed by endocytosis. At early stages, a large number of intralumenal vesicles (ILVs) are formed by the internal budding of the endosomal membrane. Late endosomes or multivesicular bodies (MVBs) are then formed through a series of changes. When MVBs fuse with the cell membrane, internal ILVs are released extracellularly as exosomes, which may then be absorbed by target cells (Maehara et al., 2021) (Figure 1).

**FIGURE 2:**
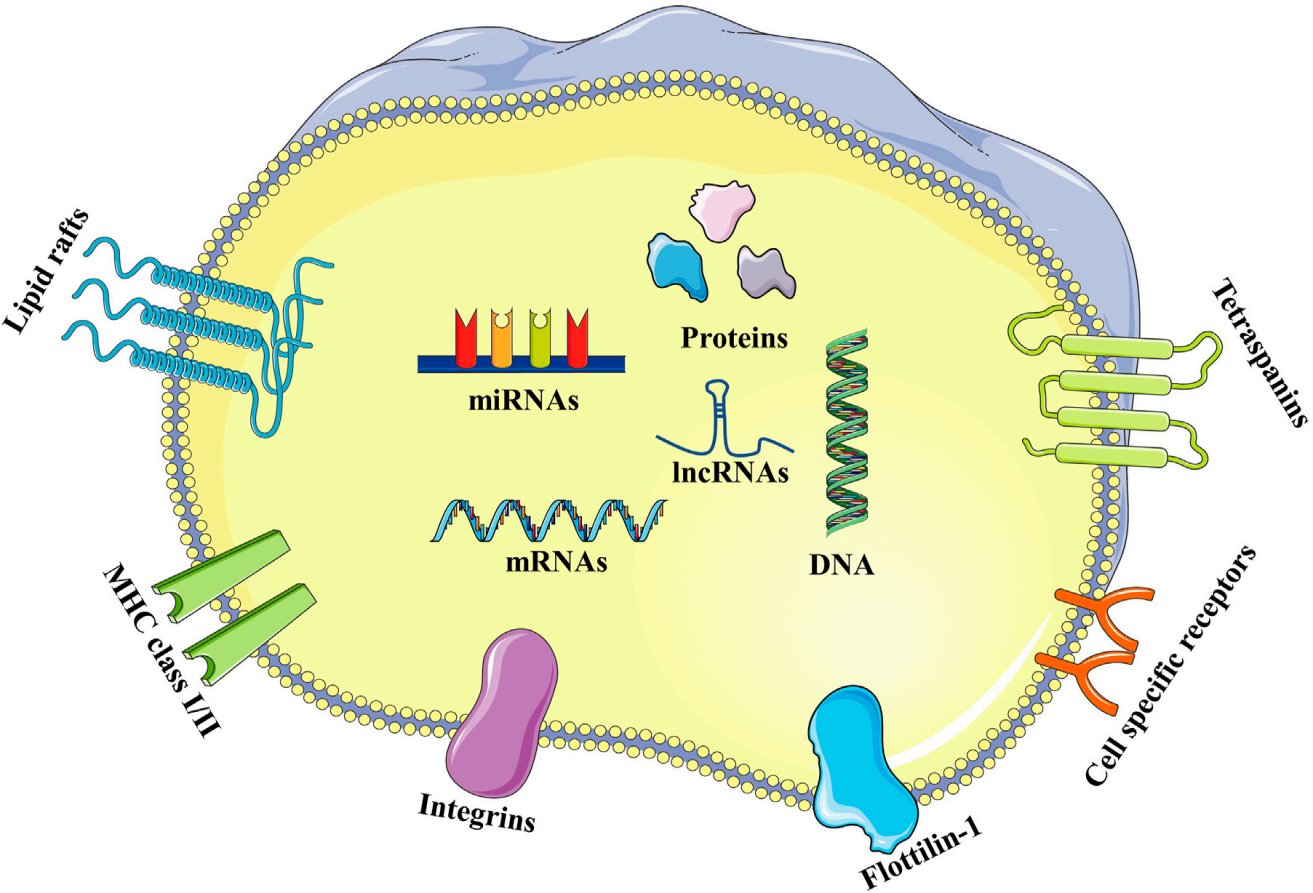
Structure of exosomes. Exosomes have a lipid bilayer membrane composed of lipid raft constituents (such as cholesterol, sphingomyelin, and ceramide), tetraspanins (such as CD9, 63 and 81), and proteins (such as integrin, cell specific receptors, major histocompatibility complex class I and class II, and flottilin-1). Proteins, lipids, and nucleic acids carried by exosomes, including DNA, mRNA, miRNA, and lncRNA.

Exosomes have a lipid bilayer membrane consisting of lipid raft constituents (such as cholesterol, sphingomyelin, and ceramide), tetraspanins (such as CD9, 63, and 81), and proteins (such as integrin, cell specific receptors, major histocompatibility complex class I and class II, and flotillin-1) (Maehara et al., 2021). Proteins, lipids, and nucleic acids are carried by exosomes, including messenger RNA (mRNA), microRNA (miRNA), and long-chain non-coding RNA (lncRNA). These molecules play essential roles in intercellular communication and the immune response (Kao and Papoutsakis, 2019) (Figure 2).

Exosomes may be isolated by multiple methods, including classical ultracentrifugation, ultrafiltration, polyethylene glycol precipitation, immunoaffinity purification, microfluidic technology, and using various commercially available kits. However, there is currently no consensus regarding the specific markers that define EV subtypes, and there is some overlap in the size of the EV subtypes. Consequently, current isolation methods cannot be used for the purification of specific EV subtypes. So, the International Society for Extracellular Vesicles (ISEV) suggests that EV subtypes should be classified according to size (<100 nm or <200 nm, small EVs or >200 nm, medium/large EVs), density (low, middle, and high), biochemical composition (CD63+/CD81+-EVs, Annexin A5-stained EVs, etc.), and origin (podocyte EVs, hypoxic EVs, large oncosomes, apoptotic bodies) (Thery et al., 2018). In this review, “exosomes” essentially refer to EVs based on the expression of typical markers without demonstration of their biogenesis, origin, and/or purity.

## 3 Therapeutic Effects of Exosomes in Osteoarthritis

### 3.1 Exosomes Promote Chondrocyte Proliferation in Osteoarthritis

Human bone marrow MSC-derived exosomes (BMSC-Exos) promote chondrocyte proliferation, which is associated with the lncRNA, KLF3-AS1. KLF3-AS1 is enriched in BMSC-Exos and after lentivirus-mediated siRNA targeting of KLF3-AS1 in BMSCs, the effect of BMSC-Exos on promoting chondrocyte proliferation was partially reversed (Liu et al., 2018a). A follow-up study revealed that BMSC-Exos with KLF3-AS1 overexpression exhibited a stronger effect on promoting chondrocyte proliferation, which was mediated by KLF3-AS1 in exosomes through the miR-206/GIT1 axis (Liu et al., 2018b). In addition, Wang et al. found that TGF-β1 promoted chondrocyte proliferation by stimulating BMSCs to secrete exosomes containing miR-135b. Furthermore, this effect was achieved by the negative regulation of Sp1 by exosomal miR-135b (Wang et al., 2018). The expression of miR-92a-3p in OA chondrocyte-derived exosomes decreased significantly, whereas BMSC-Exos with miR-92a-3p overexpression promoted chondrocyte proliferation, which was caused by exosomal miR-92a-3p inhibiting Wnt5a expression (Mao et al., 2018a). In a study on BMSC-Exos, BMSC-Exos with miR-320c overexpression had a better effect at promoting chondrocyte proliferation compared with those without miR-320c overexpression (Sun et al., 2019). Interestingly, BMSC-Exos derived from different anatomic locations exhibit different effects on chondrocyte proliferation. One study showed that exosomes secreted by polydactyly bone marrow-derived MSCs (pBMSC-Exos) have a stronger effect on promoting chondrocyte proliferation compared with conventional BMSC-Exos, which may be related to the high expression of BMP4 in pBMSCs (Zhou et al., 2020).

Exosomes derived from umbilical cord MSCs (UMSCs, UMSC-Exos) can promote chondrocyte proliferation. After interfering with lncRNA H19 in UMSCs, the role of UMSC-Exos in promoting chondrocyte proliferation was decreased, indicating that its mechanism in promoting chondrocyte proliferation is related to high H19 expression in exosomes (Yan et al., 2021a). Another study found that miR-29b-3p is a target of H19, which promotes chondrocyte proliferation by directly inhibiting miR-29b-3p, whereas the downregulation of miR-29b-3p enhances chondrocyte proliferation by negatively regulating FoxO3. This suggests that H19 from UMSC-derived exosomes participates in the regulation of chondrocyte proliferation through the miR-29b-3p/FoxO3 axis (Yan et al., 2021b). Moreover, in a hollow fiber bioreactor, exosomes derived from 3D-cultured UMSCs (3D UMSC-Exos) exhibited a strong effect at promoting chondrocyte proliferation and upregulation of TGF-β1 and Smad2/3 expression. Therefore, 3D UMSC-Exos promote chondrocyte proliferation by specifically activating the TGF-β1-dependent Smad2/3 signaling pathway (Yan and Wu, 2020).

Wang et al. (2020) demonstrated that intra-articular injection of EVs secreted by chondrogenic progenitor cells (CPCs) from MRL/MpJ superhealer mice (MRL-EVs) increased the proliferation of mouse chondrocytes. This effect was greater compared with that of EVs secreted by CPCs from control CBA mice (CBA-EVs). An analysis of the miRNA expression profiles of MRL-EVs and CBA-EVs revealed differentially expressed miRNAs involved in various biological processes, including 80 significantly up-regulated and 100 down-regulated miRNAs. Interestingly, a miR-221-3p inhibitor significantly reduced chondrocyte proliferation induced by CBA-EVs and MRL-EVs. These findings suggest that the role of CPC-secreted EVs in promoting chondrocyte proliferation may be associated with miR-221-3p; however, the underlying mechanism requires further study. The expression of miR-95-5p in exosomes derived from OA chondrocytes was found to be significantly reduced, whereas exosomes derived from primary chondrocytes with miR-95-5p overexpression promoted chondrocyte proliferation, which occurred through inhibition of HDAC2/8 expression by exosomal miR-95-5p (Mao et al., 2018b).

Exosomes derived from synovial MSCs (SMSC-Exos) promote the proliferation of chondrocytes by activating YAP but reduce the secretion of ECM components. SMSC-Exos exhibiting miR-140-5p overexpression prevent this by inhibiting RALA while promoting chondrocyte proliferation (Tao et al., 2017). Interestingly, Wang et al. demonstrated that SMSC-Exos promoted chondrocyte proliferation but did not affect ECM secretion, whereas SMSC-Exos with miR-155-5p overexpression promoted ECM secretion as well as chondrocyte proliferation (Wang et al., 2021).

In a study of fibroblast-like synoviocyte (FLS)-derived exosomes (FLS-Exos), Tan et al. found that H19 in FLS-Exos exhibiting H19 overexpression regulated the expression of TIMP2 by acting as a sponge for miR-106b-5p, thus promoting chondrocyte proliferation (Tan et al., 2020). In contrast, Zhang et al. reported that H19 inhibited chondrocyte proliferation in OA through miR-106b-5p (Zhang X. et al., 2019). In addition, synovial fibroblast (SFC)-derived exosomes (SFC-Exos) with miR-126-3p overexpression also promoted chondrocyte proliferation (Zhou et al., 2021).

Exosomes derived from platelet-rich plasma (PRP-Exos) can also promote chondrocytes proliferation. For example, Liu et al. (2019) successfully isolated and purified PRP-Exos. The results indicated that chondrocyte viability decreased following treatment of rabbit chondrocytes with IL-1β *in vitro*, whereas this effect disappeared when PRP-Exos were added. Furthermore, PRP-Exos inhibited the expression of β-catenin, Wnt5a, and RUNX2 compared with that in the IL-1β group. These findings indicate that PRP-Exos promote chondrocyte proliferation through the Wnt/β-catenin signaling pathway.

As the only existing cells in cartilage tissue, chondrocytes produce and secrete ECM to maintain the normal physiological function of the cartilage. During OA, there is an imbalance between anabolism and catabolism in the cartilage, which results in disease progression. Consequently, promoting chondrocyte proliferation may represent a treatment method to prevent OA-induced cartilage degradation.

### 3.2 Exosomes Inhibit Chondrocyte Apoptosis in Osteoarthritis

BMSC-Exos inhibit chondrocyte apoptosis in OA (Liu et al., 2018a; Cosenza et al., 2017; Zhu et al., 2018). Qi et al. successfully isolated and identified BMSC-Exos that were absorbed by chondrocytes, which inhibited IL-1β-induced chondrocyte apoptosis. This also resulted in the inhibition of p38 and ERK phosphorylation and promoted AKT phosphorylation (Qi et al., 2019). Another recent study showed that the inhibitory effect of BMSC-Exos on chondrocyte apoptosis is associated with high expression of miR-127-3p, which inhibited the expression of CDH11 (Dong et al., 2021). It was also found that IL-1β activated the Wnt/β-catenin pathway in chondrocytes and BMSC-Exos inhibited this activity, whereas overexpression of CDH11 weakened the inhibitory effect of BMSC-Exos on Wnt/β-catenin signaling. Therefore, miR-127-3p carried by BMSC-Exos blocks the activation of the Wnt/β-catenin pathway by inhibiting CDH11 expression in chondrocytes, thus inhibiting apoptosis (Dong et al., 2021). Additionally, BMSC-Exos with KLF3-AS1 overexpression also significantly inhibited chondrocyte apoptosis (Liu et al., 2018b). Luciferase reporter gene assays revealed that KLF3-AS1 sponges miR-206 to promote the expression of GIT1, whereas overexpression of miR-206 or knockdown of GIT1 reverses the KLF3-AS1-mediated inhibition of chondrocyte apoptosis. Therefore, exosomal KLF3-AS1 inhibits chondrocyte apoptosis through the miR-206/GIT1 axis. Lin et al. reported that DPSC-Exos with miR-140-5p overexpression inhibited IL-1β-induced chondrocyte apoptosis compared with controls (Lin et al., 2021), which may occur through the regulation of apoptosis-related proteins.

It has been reported that curcumin delays OA progression; however, its underlying mechanism remains unclear. Qiu et al. reported that curcumin-treated BMSC-Exos inhibited IL-1β-induced chondrocyte apoptosis (Qiu et al., 2020), which occurred through the exosomal regulation of the miR-124/NF-kB and miR-143/ROCK1/TLR9 pathways.

The miR-129-5p levels in synovial cells of OA patients are significantly reduced and exhibit a negative correlation with high mobility group protein-1 (HMGB1) levels, which are significantly increased. In addition, IL-1β significantly decreased the expression of miR-129-5p in chondrocytes and up-regulated HMGB1 levels in chondrocytes. In a bioinformatics analysis, HMGB1 was identified as a target of miR-129-5p (Qiu et al., 2021). Further studies showed that SMSC-Exo with miR-129-5p overexpression attenuated IL-1β-induced chondrocyte apoptosis. In contrast, SMSC-Exo with miR-129-5p knockdown enhanced IL-1β-induced apoptosis. Therefore, miR-129-5p from SMSC-Exo targets HMGB1 and inhibits chondrocyte apoptosis in OA (Qiu et al., 2021). Wang et al. showed that SMSC-Exo with miR-129-5p overexpression also inhibited chondrocyte apoptosis in OA, which occurred through targeted inhibition of exosomal miR-155-5p on RUNX2 (Wang et al., 2021). Recent studies have demonstrated that the expression of miR-126-3p in SFC-Exos from OA patients was significantly decreased, whereas SFC-Exos with miR-126-3p overexpression inhibited the chondrocyte apoptosis in OA (Zhou et al., 2021).

Zhang et al. demonstrated that exosomes derived from human embryonic MSC (EMSC-Exos) inhibited chondrocyte apoptosis in OA by activating the adenosine-dependent AKT and ERK pathways, whereas inhibitors of AKT/ERK phosphorylation decreased the exosome-mediated inhibitory effect (Zhang et al., 2016). The same group also demonstrated in a rat model of temporomandibular joint arthritis that the inhibition of chondrocyte apoptosis mediated by EMSC-Exos was the result of adenosine activation of the AKT, ERK, and AMPK signaling pathways (Zhang S. et al., 2019).

Moreover, infrapatellar fat pad MSC (IPFPMSC)-derived exosomes (IPFPMSC-Exos) inhibited IL-1β-induced chondrocyte apoptosis, which occurred through miR-100-5p in exosomes inhibiting the mTOR pathway and promoting autophagy (Wu et al., 2019). Zhao et al. reported that adipose MSC (ADMSC)-derived exosomes (ADMSC-Exos) significantly inhibited H_2_O_2_-induced chondrocyte apoptosis (Zhao et al., 2020). In addition, whereas UMSC-Exos promote chondrocyte proliferation, they also inhibit chondrocyte apoptosis through the H19/miR-29b-3p/FoxO3 axis (Yan et al., 2021a; Yan et al., 2021b) or by regulating the Smad2/3 signaling pathway (Yan and Wu, 2020).

Although it is unknown whether chondrocyte apoptosis is the cause or result of OA progression (Charlier et al., 2016), chondrocyte apoptosis aggravates cartilage degradation and exhibits a positive correlation with the severity of OA (Thomas et al., 2007). Therefore, inhibition of chondrocyte apoptosis represents a potential treatment strategy to prevent OA-induced cartilage degradation.

### 3.3 Exosomes Inhibit Chondrocyte Inflammatory in Osteoarthritis

Exosomes inhibit the expression of pro-inflammatory factors or promote their release. In a chondrocyte model of IL-1β-induced inflammatory injury, exosomes from various sources inhibited the expression of several pro-inflammatory cytokines (Cosenza et al., 2017; Jin et al., 2020a; Liu C. et al., 2020; Liu X. et al., 2020; Zhao et al., 2020; Qiu et al., 2021; Zhou et al., 2021). Jin et al. showed that BMSC-Exos exhibiting miR-26a-5p overexpression inhibited the IL-1β-induced expression of pro-inflammatory cytokines in SFCs, including IL-6, IL-8, and TNF-α. In addition, miR-26a-5p inhibited PTGS2, whereas the overexpression of PTGS2 reversed its inhibitory effect on inflammation. Thus, exosomal miR-26a-5p reduces inflammatory damage to SFCs during OA by inhibiting the expression of PTGS2 (Jin et al., 2020b). Luo et al. showed that miR-100-5p was highly expressed in human exfoliated deciduous teeth (SHED) stem cell-derived exosomes (SHED-Exos). Treatment of IL-1β-induced OA chondrocytes with SHED-Exos inhibited the expression of IL-6 and IL-8. Moreover, a luciferase reporter gene assay showed that miR-100-5p directly targeted and inhibited mTOR expression. Furthermore, the expression of pro-inflammatory cytokines was inhibited following treatment of OA chondrocytes with rapamycin, an mTOR pathway inhibitor. Therefore, SHED-Exos inhibits chondrocyte inflammation through miR-100-5p inhibition of mTOR expression (Luo et al., 2019).

The symptoms associated with inflammatory injury during OA include swelling and pain (Castrogiovanni et al., 2019; Di Rosa et al., 2019). Therefore, ameliorating these effects is another manifestation of the anti-inflammatory activity of exosomes. For example, in a collagen-induced arthritis model, intravenous injection of BMSC-Exos resulted in a decrease in the degree of mouse paw swelling compared with the control group (Cosenza et al., 2018). In addition, He et al. reported that BMSC-Exos inhibited the expression of the pro-inflammatory cytokines, IL-1β, IL-6, and TNF-α, in the sera of OA rats, which was induced by sodium iodoacetate, whereas expression of the anti-inflammatory cytokine IL-10 was increased. This was accompanied by a reduction of pain in OA rats (He et al., 2020). Moreover, EMSC-Exos inhibited pain in rats with early temporomandibular joint arthritis and reduced inflammatory damage (Zhang S. et al., 2019). Finally, exosomes derived from amniotic fluid stem cells (AFSC) increased the pain tolerance of rats in which OA was induced by sodium iodoacetate (Zavatti et al., 2020).

In OA, M1 macrophages present antigens that activate the Th1 immune response and promote inflammation, whereas the Th2 response induced by M2 macrophages exhibits an anti-inflammatory effect and promotes cartilage repair by inhibiting inflammation (Chen Y. et al., 2020). Consequently, another anti-inflammatory effect of exosomes is the reduction of M1 macrophages, an increase of M2 macrophages, or the promotion of macrophage polarization from the M1 to the M2 type. Zhang et al. found that injection of BMSC-Exos decreased the expression of pro-inflammatory cytokines and promoted the release of anti-inflammatory cytokines in the serum of OA rats. They also demonstrated that following BMSC-Exo treatment, the number of M1 macrophages in the rat synovial tissue decreased significantly, whereas that of M2 macrophages increased. This finding was confirmed *in vitro* using an LPS-induced RAW264.7 inflammatory cell model. Therefore, BMSC-Exos promote macrophage polarization from the M1 to the M2 type and relieve inflammation (Zhang et al., 2020). Moreover, a 3D-printed ECM/gelatin methacrylate/exosome scaffold had no toxic effect in rats and promoted the polarization of synovial macrophages to the M2 type (Chen et al., 2019).

Recent studies have shown that miR-135b is highly expressed in TGF-β1-stimulated BMSC-Exos (BMSC-Exos^TGF-β1^), and they decrease the expression of serum pro-inflammatory cytokines and M1 polarization of synovial macrophages in OA rats. Furthermore, they promote the polarization of M2 synovial macrophages, which may be reversed by miR-135b inhibitors. A bioinformatics analysis in conjunction with luciferase reporter gene assays demonstrated that MAPK6 is inhibited by miR-135b, whereas overexpression of MAPK6 reversed the promoting effect of BMSCs-Exos^TGF-β1^ on the polarization of M2 synovial macrophages. These results indicate that miR-135b, which is highly expressed in BMSCs-Exos^TGF-β1^, promotes the M2 polarization of synovial macrophages by inhibiting MAPK6 expression, thereby inhibiting chondrocyte inflammation (Wang and Xu, 2021). In addition, exosomes from other sources also exhibited the same effect (Zhang et al., 2018; Woo et al., 2020; Zheng et al., 2019).

Finally, as a classic inflammatory signaling pathway, NF-κB is involved in the inflammatory damage of the cartilage and synovium in OA (Lepetsos et al., 2019). Therefore, inhibiting the activation of the NF-κB pathway is another anti-inflammatory effect of exosomes. Bone marrow MSC-derived EVs (BMSC-EVs) down-regulate TNF-α-induced expression of the pro-inflammatory cytokines, IL-1α, IL-1β, IL-6, IL-8, and IL-17, in OA chondrocytes. Another study demonstrated that BMSC-EVs inhibit the TNF-α-induced transfer of p65 from the cytoplasm to the nucleus in OA chondrocytes and inhibit IκBα phosphorylation (an inhibitory subunit of NFκB). Therefore, BMSC-EVs inhibit TNF-α-induced activation of the NF-κB pathway (Vonk et al., 2018). Moreover, ADMSC-Exos inhibit the IL-1β-induced binding of p65 to DNA in the nucleus of OA chondrocytes, thus inhibiting the activation of the NF-κB signaling pathway (Tofino-Vian et al., 2018).

Recent studies have shown that in an SW982 human synovial cell line-induced OA model treated with IL-1β and TNF-α, BMSC-Exos treated with IL-1β (BMSC-Exos^IL-1β^) resulted in a stronger inhibitory effect on the expression of pro-inflammatory cytokines compared with the untreated controls. In addition, a higher level of miR-147b was found in BMSC-Exos^IL-1β^, whereas miR-147b mimics significantly inhibited the expression of pro-inflammatory cytokines. Both BMSC-Exos^IL-1β^ and miR-147b mimics inhibited IL-1β and TNF-α-induced IκBα downregulation. These results indicate that the anti-inflammatory effects of BMSC-Exos^IL-1β^ are mediated by miR-147b, which is involved in the inhibition of the NF-κB pathway (Kim M. et al., 2021).

Even in the early stage of OA, synovitis is a hallmark of this disease (Scanzello and Goldring, 2012). Many cytokines and chemokines lead to the further development of OA. As a result, anti-inflammatory therapies are indispensable for the treatment of OA.

### 3.4 Exosomes Protect Cartilage Extracellular Matrix in Osteoarthritis

Studies have shown that BMSC-Exos protect cartilage from OA degradation by increasing the expression of chondrocyte markers, such as aggrecan (ACAN) and collagen II (COL2A1), and reducing the levels of catabolic markers, such as matrix metalloproteinases (MMPs) and a disintegrin and metalloproteinase with thrombospondin type 1 motifs, member 5 (ADAMTS5) (Cosenza et al., 2017; Zhu et al., 2018; He et al., 2020; Chen et al., 2019). With respect to the underlying mechanism, Liu et al. reported that the therapeutic effect of BMSC-Exos may be associated with KLF3-AS1 expression in these exosomes. Additionally, treatment of OA chondrocytes with BMSC-Exos with KLF3-AS1 knockdown reversed the protective effects on chondrocytes (Liu et al., 2018a). Huang et al. demonstrated that BMSC-Exos inhibited the degradation of cartilage ECM in OA through inhibition of CDH11 expression by miR-127-3p, thus blocking the activation of the Wnt/β-catenin signaling pathway (Dong et al., 2021).

Another study demonstrated that exosomal miR-320c (Sun et al., 2019), miR-92a-3p (Mao et al., 2018a), KLF3-AS1 (Liu et al., 2018b), and miR-136-5p (Chen X. et al., 2020) from transfected BMSCs, inhibited cartilage ECM degradation during OA. Of these, the expression of miR-136-5p was decreased in OA cartilage tissue and a luciferase reporter gene assay revealed that the expression of its target gene, ELF3, was increased in OA cartilage tissue. However, high ELF3 expression reversed the effect of miR-136-5p in promoting the synthesis of cartilage ECM, indicating that exosomal miR-136-5p promotes the synthesis of cartilage ECM by inhibiting ELF3 in OA (Chen X. et al., 2020). In IL-1β-treated human chondrocytes, DPSC-Exos enhanced the expression of chondrocyte related mRNAs, including ACAN, COL2A1, and Sox9, whereas DPSC-Exos with miR-140-5p overexpression significantly enhanced this effect (Lin et al., 2021).

EMSC-Exos inhibit the IL-1β-induced degradation of cartilage ECM by increasing COL2A1 synthesis and decreasing ADAMTS5 expression (Wang et al., 2017). Zhang et al. reported that EMSC-Exos promoted cartilage ECM synthesis by activating the adenosine-dependent AKT and ERK pathways in OA (Zhang et al., 2018). In addition, ADMSC-Evs inhibited the IL-1β-induced production of MMP1/3/13 and ADAMTS5 and increased the expression of COL2A1 in OA chondrocytes (Woo et al., 2020). Tofino-Vian et al. attributed the protective effects of ADMSC-Exos on chondrocytes to the inhibition of NF-κB binding AP-1 sites in DNA (Tofino-Vian et al., 2018). MiR-100-5p regulates AKT/mTOR signal transduction by targeting mTOR in various diseases (Ye et al., 2015). Interestingly, high expression of miR-100-5p was found in both IPFPMSC-Exos and SHED-Exos and reduced IL-1β-induced MMP13 and ADAMTS5 expression in OA chondrocytes by inhibiting mTOR signaling (Luo et al., 2019; Wu et al., 2019).

High expression of H19 in UMSC-Exos inhibited IL-1β-induced MMP13 and ADAMTS5 upregulation as well as ACAN and COL2A1 downregulation in OA chondrocytes (Yan et al., 2021a). Furthermore, it was shown that H19 in UMSC-Exos participates in the synthesis of cartilage ECM mediated by UMSC-Exos through the miR-29b-3p/FoxO3 axis in OA (Yan et al., 2021b). UMSC-derived exosomes, cultured in 3D using a hollow fiber bioreactor, alleviated the degradation of cartilage ECM by specifically activating the TGF-β1-dependent Smad2/3 pathway in OA (Yan and Wu, 2020).

In addition to MSCs, the therapeutic effect of primary chondrocyte-derived exosomes on cartilage ECM has been demonstrated (Zheng et al., 2019; Liu X. et al., 2020), although the underlying mechanism has not been determined. Liu X. et al., 2020 showed that EVs derived from IL-1β-treated chondrocytes stimulated catabolic events in chondrocytes as evidenced by increased expression of the catabolic markers, cyclooxygenase-2 (cox-2), IL-6, inducible nitric oxide synthase (iNOS), and MMP13, and decreased expression of the articular cartilage markers, ACAN and COL2A1. EVs derived from vehicle-treated chondrocytes exhibited reduced cox-2, IL-6, iNOS, and MMP13 mRNA levels and increased expression of articular cartilage markers, ACAN and COL2A1 in IL-1β-treated chondrocytes. In addition, EVs derived from IL-1β-treated chondrocytes inhibited chondrogenesis of precursor cells, whereas EVs derived from vehicle-treated chondrocytes stimulated chondrogenesis.

Furthermore, Zheng et al. (2019) isolated exosomes from primary chondrocytes cultured under normal (D0) and inflammatory conditions induced by IL-1β and performed proteomics analysis of the exosomes. They found that there were more proteins associated with the mitochondria and involved in the immune system in D0 exosomes. The intra-articular injection of D0 exosomes prevented the development of OA. This indicates that D0 exosomes restore mitochondrial dysfunction and promote macrophage polarization to the M2 type. In another study, primary chondrocyte-derived exosomes exhibiting miR-95-5p overexpression directly targeted HDAC2/8 and significantly up-regulated the expression of ACAN, COL2A1, collagen alpha-1(IX) (COL9A1), and cartilage oligomeric matrix protein, and down-regulated the expression of collagen alpha-1(X) (COL10A1), and MMP13 in OA chondrocytes (Mao et al., 2018b). Thus, these findings indicate that a thorough understanding of exosomesderived from chondrocytes may lead to novel therapeutic strategies for OA treatment.

Studies have shown that SMSC-Exos do not promote or reduce the secretion of cartilage ECM in OA (Tao et al., 2017; Wang et al., 2021). However, exosomal miR-140-5p and miR-155-5p from transfected SMSCs up-regulated the expression of COL2A1 and sry-related high mobility group box 9tbox9 (SOX9) in OA chondrocytes by inhibiting RALA and RUNX2, respectively. Another study demonstrated that SMSC-Exos promoted IL-1β-induced upregulation of COL2A1 and downregulation of MMP13 in OA chondrocytes (Qiu et al., 2021). Furthermore, exosomal miR-129-5p from SMSCs enhanced this effect by inhibiting HMGB1. Finally, FLS-Exos with H19 overexpression inhibited IL-1β-induced degradation of cartilage ECM, which was mediated by the H19/miR-106b-5p/TIMP2 axis in OA (Tan et al., 2020).

Degradation of cartilage ECM is an important characteristic of OA and primarily manifests as an inhibited synthesis of ACAN and COL2A1, and up-regulation of MMPs and ADAMTS5. Therefore, a major goal of OA treatment is to limit the degradation of cartilage ECM.

### 3.5 Preclinical *In Vivo* Studies of Exosomes in Osteoarthritis

Preclinical *in vivo* studies are important in evaluating the safety, efficacy, and clinical translation of exosomes for the treatment of OA. Zhang et al. (2016) reported that the effects observed following exosome treatment showed enhanced gross appearance and improved histological scores compared with that of phosphate buffered saline. At 12 weeks, the effects associated with exosome treatment indicated complete cartilage and subchondral bone repair, which was characterized by hyaline cartilage with good surface regularity, complete binding to adjacent cartilage, and ECM deposition. In addition, no detrimental response was observed in the animals.

Apart from a gross morphological assessment, Wong et al. (2020) also performed a biomechanical assessment of regenerated cartilage tissue. Compared with the effects of hyaluronic acid (HA) alone, exosomes and HA treatment improved mechanical properties (such as Young’s modulus and stiffness). At 12 weeks, the newly repaired tissue was obtained following treatment with exosomes and HA, which was superior mechanically and structurally to that obtained by HA treatment alone, whereas similar mechanical properties were observed in the adjacent native cartilage. These findings suggest that exosomes promote the repair of cartilage tissue morphology and contribute to the improvement of mechanical properties.

Tao et al. (2017) demonstrated that SMSC-Exos exhibiting miR-140-5p overexpression (SMSC-140-Exos) enhanced the proliferation of chondrocytes without damaging the *in vitro* secretion of ECM. They subsequently verified the potential of exosomes in preventing OA in a rat model. Compared with the OA group, the joint wear and cartilage ECM loss were both improved in the OA SMSC-Exo and OA+SMSC-140-Exo groups. In addition, the expression of COL2A1 and ACAN in cartilage was increased, whereas the expression of type I collagen was decreased. Notably, the OA+SMSC-140-Exo group exhibited a superior therapeutic effect compared with the OA+SMSC-Exo group. These findings suggest a role for SMSC-Exos in repairing cartilage associated with miR-140-5p expression.

The research on OA treatment with exosomes remains limited to cell and animal experiments. In addition, the animal experiments are limited to mice, rats, and rabbits. Therefore, these studies need to be expanded to large animals and eventually, the clinical and radiological effects of exosomes on OA should be evaluated in clinical trials.

## 4 Discussion and Perspective

It was originally thought that exosomes were a useless metabolic byproduct. However, it is now recognized that exosomes carry proteins, lipids, and various nucleic acids (mRNA, miRNA, and lncRNA) that play important roles in intercellular communication and the immune response (Kao and Papoutsakis, 2019). Exosomes exist in various body fluids and cells. Exosomes from different sources contain a variety of RNA and protein components, which can be used as biomarkers for the early diagnosis of disease and carriers for therapeutics. Accordingly, exosomes have gained interest in research areas that include disease biomarkers, disease mechanisms, and drug development.

Recent studies have demonstrated that exosomes from various cells have positive regulatory effects on chondrocyte proliferation, apoptosis, inflammation, and ECM synthesis in OA. Nevertheless, there are many issues that need to be addressed. First, exosomes from various cell types are heterogeneous (Reiner et al., 2017). They contain different biomolecules including proteins, nucleic acids, and lipids, resulting in different therapeutic effects. However, a few studies have compared the therapeutic effects of exosomes from different sources on chondrocytes in OA to select the optimal cell source for treatment. Only one study reported that exosomes derived from induced pluripotent stem cell-derived MSCs (iMSC-Exos) exhibited a stronger effect than SMSC-Exos on promoting chondrocyte proliferation (Zhu et al., 2017) and BMSC-Exos from polydactyly tissue had a stronger effect at promoting chondrocyte proliferation compared with BMSC-Exos from conventional tissue (Zhou et al., 2020).

Additionally, exosomes derived from allogeneic donor cells exhibit different effects after pretreatment, which are mainly manifested as enhanced therapeutic effects. For example, Chen et al. demonstrated that BMSC-Exos exhibiting miR-136-5p overexpression had a better inhibitory effect on cartilage degradation compared with BMSC-Exos without miR-136-5p overexpression (Chen X. et al., 2020). Moreover, Wang et al. showed that SMSC-Exos with miR-155-5p overexpression exhibited a stronger effect in promoting chondrocyte proliferation and inhibiting chondrocyte apoptosis compared with control SMSC-Exos. They also enhanced the secretion of cartilage ECM by targeting and inhibiting RUNX2, whereas SMSC-Exos without miR-155-5p overexpression did not (Wang et al., 2021). Interestingly, 3D UMSC-Exos exhibited better effects on chondrocyte proliferation, migration, and ECM synthesis, as well as inhibiting chondrocyte apoptosis compared with those cultured using routine 2D-culture techniques (Yan and Wu, 2020). However, it should be noted that these results are from one report and should be verified through additional studies.

Despite their extensive presence in various body fluids and cells, exosomes derived from MSCs appear to be preferred by researchers. Thus far, the use of MSCs represents a promising treatment strategy for the treatment of OA, particularly because MSCs can migrate to the injury site, differentiate into cells of the appropriate phenotype, and synthesize ECM. However, MSCs also have several limitations for OA treatment. For example, they exhibit a short lifespan after transplantation into target organs and disappear in just a few days (Bunnell et al., 2008; Heldring et al., 2015; Paschos and Sennett, 2017). In addition, the immune tolerance of the target organs or target cells resulting from MSCs treatment may increase the risk of developing tumors (Lalu et al., 2012). MSCs may exhibit pro-inflammatory activity at the initial stage of transplantation to activate the innate immune system and subsequently exhibit anti-inflammatory activity. During this process, the decline in MSC levels greatly reduces their effectiveness. (Le Blanc and Mougiakakos, 2012).

Exosomes derived from MSCs share the same biological properties as MSCs. In particular, they have significant advantages because of their small size and low immunogenicity. In addition, as a cell-free therapeutic approach, MSC-derived exosomes may avoid issues associated with direct cell injection. Furthermore, the long-term storage of exosomes is adequate. Storage at −20°C or repeated freezing and thawing does not affect exosome size, whereas long-term storage at −80°C preserved high activity (Sokolova et al., 2011; Lorincz et al., 2014). Therefore, MSC-derived exosomes represent a promising treatment strategy for OA.

Generally, MSCs may be of more research value as a source for OA treatment. Certainly, such MSCs should meet the minimum requirements for MSCs established by the International Society for Cell and Gene Therapy. (Dominici et al., 2006). However, large experiments are required for the discovery of other MSC sources. The efficacy of MSC-derived exosomes on OA, as well as the difficulty of obtaining donor-derived MSCs, must be considered. Moreover, another issue is whether the MSCs should be modified to obtain exosomes that carry specific cargos for satisfying specific treatment protocols. Once the appropriate cell source of exosomes for treating OA has been established, maintaining the consistency of the cell sources will be a challenge. However, immortalized monoclonal cell lines derived from MSCs can solve this problem. For example, exosomes from myc-mediated immortalized MSCs show cardioprotective effects (Chen et al., 2011). However, immortalization may pose a health risk Table 1.

**TABLE 1:** Summary of the roles of exosomes in OA chondrocytes.

Therapeutic effects	Origin	Inclusions	Targets	Reference
Promote chondrocyte proliferation	BMSC	lncRNA KLF3-AS1	-	Liu et al. (2018a)
	BMSC with KLF3-AS1 overexpression	lncRNA KLF3-AS1	KLF3-AS1/miR-206/GIT1 axis	Liu et al. (2018b)
	BMSC with TGF-β1 treatment	miR-135b	Sp 1	Wang et al. (2018)
	BMSC with miR-92a-3p overexpression	miR-92a-3p	Wnt5a	Mao et al. (2018a)
	BMSC with miR-320c overexpression	miR-320c	-	Sun et al. (2019)
	pBMSC	BMP4	-	Zhou et al. (2020)
	UMSC	lncRNA H19	-	Yan et al. (2021a)
	UMSC	lncRNA H19	H19/miR-29b-3p/FoxO3 axis	Yan et al. (2021b)
	UMSC	-	TGF-β1-dependent Smad2/3 pathway	Yan and Wu. (2020)
	CPC	miR-221-3p	-	Wang et al. (2020)
	Chondrocyte with miR-95-5p overexpression	miR-95-5p	HDAC2/8	Mao et al. (2018b)
	SMSC with miR-140-5p overexpression	miR-140-5p	RalA	Tao et al. (2017)
	SMSC with miR-155-5p overexpression	miR-155-5p	Runx2	Wang et al. (2021)
	FLS with H19 overexpression	lncRNA H19	H19/miR-106b-5p/TIMP2 axis	Tan et al. (2020)
	SFC with miR-126-3p overexpression	miR-126-3p		Zhou et al. (2021)
	PRP	-	Wnt/β-catenin pathway	Liu et al. (2019)
Inhibit chondrocyte apoptosis	BMSC	lncRNA KLF3-AS1	-	Liu et al. (2018a)
	BMSC	-	-	Cosenza et al. (2017)
	BMSC	-	-	Zhang et al. (2016)
	BMSC	-	p38/ERK/AKT pathway	Qi et al. (2019)
	BMSC	miR-127-3p	CDH11-mediated Wnt/β-catenin pathway	Dong et al. (2021)
	BMSC with KLF3-AS1 overexpression	lncRNA KLF3-AS1	KLF3-AS1/miR-206/GIT1 axis	Liu et al. (2018b)
	BMSC with curcumin treatment	miR-124/miR-143	miR-124/NF-κB and miR-143/ROCK1/TLR9 pathway	Qiu et al. (2020)
	DPSC with miR-140-5p overexpression	miR-140-5p	Apoptosis-related proteins	Lin et al. (2021)
	SMSC with miR-129-5p overexpression	miR-129-5p	HMGB1	Qiu et al. (2021)
	SMSC with miR-155-5p overexpression	miR-155-5p	Runx2	Wang et al. (2021)
	SFC with miR-126-3p overexpression	miR-126-3p	-	Zhou et al. (2021)
	EMSC	-	Adenosine-dependent AKT/ERK pathway	Zhang et al. (2016)
	EMSC	-	Adenosine-dependent AKT/ERK/AMPK pathway	Zhang S et al. (2019)
	IPFPMSC	miR-100-5p	mTOR	Wu et al. (2019)
	ADMSC	-	miR-145/miR-221	Zhao et al. (2020)
	UMSC	lncRNA H19	-	Yan et al. (2021a)
	UMSC	lncRNA H19	H19/miR-29b-3p/FoxO3 axis	Yan et al. (2021b)
	UMSC	-	TGF-β1-dependent Smad2/3 pathway	Yan and Wu. (2020)
Inhibit chondrocyte inflammation	BMSC with Kartogenin treatment	-	-	Liu C et al. (2020)
	SFC with miR-126-3p overexpression	miR-126-3p	-	Zhou et al. (2021)
	BMSC	-	-	Cosenza et al. (2017)
	ADMSC	-	miR-145/miR-221	Zhao et al. (2020)
	SMSC with miR-129-5p overexpression	miR-129-5p	HMGB1	Qiu et al. (2021)
	BMSC	miR-9-5p	Syndecan-1	Jin et al. (2020a)
	Chondrocyte	-	-	Liu X et al. (2020)
	BMSC with miR-26a-5p overexpression	miR-26a-5p	PTGS2	Jin et al. (2020b)
	SHED	miR-100-5p	mTOR	Luo et al. (2019)
	BMSC	-	T/B lymphocytes	Cosenza et al. (2018)
	BMSC	-	-	He et al. (2020)
	EMSC	-	Adenosine-dependent AKT/ERK/AMPK pathway	Zhang S et al. (2019)
	AFSC	-	-	Zavatti et al. (2020)
	BMSC	-	-	Zhang et al. (2020)
	BMSC	-	-	Chen et al. (2019)
	BMSC with TGF-β1 treatment	miR-135b	MAPK6	Wang and Xu. (2021)
	EMSC	-	Adenosine-dependent AKT/ERK pathway	Zhang et al. (2016)
	ADMSC	-	-	Woo et al. (2020)
	Chondrocyte	-	-	Zheng et al. (2019)
	BMSC	-	NF-κB pathway	Vonk et al. (2018)
	ADMSC	-	NF-κB/AP-1	Tofino-Vian et al. (2018)
	BMSC with IL-1β treatment	miR-147b	NF-κB pathway	Kim M et al. (2021)
Protect Cartilage ECM	BMSC	-	-	Cosenza et al. (2017)
	BMSC	-	-	Zhang et al. (2016)
	BMSC	-	-	He et al. (2020)
	BMSC	-	-	Chen et al. (2019)
	BMSC	lncRNA KLF3-AS1	-	Liu et al. (2018a)
	BMSC	miR-127-3p	CDH11-mediated Wnt/β-catenin pathway	Dong et al. (2021)
	BMSC with miR-320c overexpression	miR-320c	-	Sun et al. (2019)
	BMSC with miR-92a-3p overexpression	miR-92a-3p	Wnt5a	Mao et al. (2018a)
	BMSC with KLF3-AS1 overexpression	lncRNA KLF3-AS1	KLF3-AS1/miR-206/GIT1 axis	Liu et al. (2018b)
	BMSC with miR-136-5p overexpression	miR-136-5p	ELF3	Chen Y et al. (2020)
	DPSC with miR-140-5p overexpression	miR-140-5p	Apoptosis-related proteins	Lin et al. (2021)
	EMSC	-	-	Wang et al. (2017)
	EMSC	-	Adenosine-dependent AKT/ERK pathway	Zhang et al. (2016)
	ADMSC	-	-	Woo et al. (2020)
	ADMSC	-	NF-κB/AP-1	Tofino-Vian et al. (2018)
	IPFPMSC	miR-100-5p	mTOR	Wu et al. (2019)
	SHED	miR-100-5p	mTOR	Luo et al. (2019)
	UMSC	lncRNA H19	-	Yan et al. (2021a)
	UMSC	lncRNA H19	H19/miR-29b-3p/FoxO3 axis	Yan et al. (2021b)
	UMSC	-	TGF-β1-dependent Smad2/3 pathway	Yan and Wu. (2020)
	Chondrocyte	-	-	Liu X et al. (2020)
	Chondrocyte	-	-	Zheng et al. (2019)
	Chondrocyte with miR-95-5p overexpression	miR-95-5p	HDAC2/8	Mao et al. (2018b)
	SMSC with miR-140-5p overexpression	miR-140-5p	RalA	Tao et al. (2017)
	SMSC with miR-155-5p overexpression	miR-155-5p	RUNX2	Wang et al. (2021)
	SMSC with miR-129-5p overexpression	miR-129-5p	HMGB1	Qiu et al. (2021)
	FLS with H19 overexpression	lncRNA H19	H19/miR-106b-5p/TIMP2 axis	Tan et al. (2020)

Exosomes exhibit complex characteristics including the size and variety of active substances that are transported. In addition to the exosome source, it is important to evaluate these characteristics thoroughly. Therefore, it will be a challenge to establish unified quality standards for the production process. During the production of exosomes, the quality of the donor cells, their components, and the management of the production process must be established. Currently, there is an absence of a unified standard established for the clinical production and quality control of exosome-based therapy. Although the ISEV proposed “Minimal Information for Studies of Extracellular Vesicles” guidelines in 2014, the updated version from 2018 provides a comprehensive and evolving set of guidelines for studying EVs, including exosomes (Lener et al., 2015; Thery et al., 2018). Strict quality control should be implemented to ensure the quality, efficacy, and safety of exosome treatment.

Currently, the biological properties of exosomes have not been clearly defined. Furthermore, the donor cells, enrichment and purification methods, doses, and preclinical animal models used by researchers vary widely, thus the published data lack comparability. Therefore, assay standards for evaluating the potency of exosomes should be established across laboratories to better compare the data obtained from different studies and provide a basis for the translation of exosome therapy for OA. The potency of exosomes created by diverse protocols should be tested using a set of quantitative parameters. Exosomes with the ideal potency measurements may be selected for treating OA. Specifically, measuring the biological activity of the attributes most relevant to the regulation of OA pathology will serve as a criterion for measuring the potency of exosomes manufactured by various protocols (Gimona et al., 2021). Clearly, a better understanding of the biological properties of exosomes and the standardized methods for enrichment, purification, characterization, and development of potency assays will facilitate the clinical translation of exosomes.

## 5 Conclusion

In this review, we summarized the important roles of exosomes in the proliferation, apoptosis, inflammation, and ECM synthesis of OA chondrocytes and their molecular mechanisms. The primary role of exosomes is to promote chondrocyte proliferation and ECM synthesis, as well as inhibit chondrocyte apoptosis and damage from inflammation. The pathogenesis of OA is complex and there is currently no satisfactory treatment method. However, existing studies have demonstrated that exosomes represent a novel therapeutic strategy for OA, especially those derived from MSCs. There has been significant progress in the field of exosome research, improvement in exosome isolation and storage technology, and the establishment of standardized protocols. Accordingly, this will significantly accelerate the development of new strategies for the exosome-based treatment of OA.
